# A Molecular Signature of Proteinuria in Glomerulonephritis

**DOI:** 10.1371/journal.pone.0013451

**Published:** 2010-10-18

**Authors:** Heather N. Reich, David Tritchler, Daniel C. Cattran, Andrew M. Herzenberg, Felix Eichinger, Anissa Boucherot, Anna Henger, Celine C. Berthier, Viji Nair, Clemens D. Cohen, James W. Scholey, Matthias Kretzler

**Affiliations:** 1 Division of Nephrology, University Health Network, University of Toronto, Toronto, Ontario, Canada; 2 The Toronto Glomerulonephritis Registry, Toronto, Ontario, Canada; 3 Division of Epidemiology and Statistics, Ontario Cancer Institute, and University of Toronto, Toronto, Ontario, Canada; 4 Department of Laboratory Medicine and Pathology, University Health Network, University of Toronto, Toronto, Ontario, Canada; 5 Department of Internal Medicine, University of Michigan, Ann Arbor, Michigan, United States of America; 6 Division of Nephrology and Institute of Physiology, University Hospital Zurich, Zurich, Switzerland; University of Minnesota, United States of America

## Abstract

Proteinuria is the most important predictor of outcome in glomerulonephritis and experimental data suggest that the tubular cell response to proteinuria is an important determinant of progressive fibrosis in the kidney. However, it is unclear whether proteinuria is a marker of disease severity or has a direct effect on tubular cells in the kidneys of patients with glomerulonephritis. Accordingly we studied an *in vitro* model of proteinuria, and identified 231 “albumin-regulated genes” differentially expressed by primary human kidney tubular epithelial cells exposed to albumin. We translated these findings to human disease by studying mRNA levels of these genes in the tubulo-interstitial compartment of kidney biopsies from patients with IgA nephropathy using microarrays. Biopsies from patients with IgAN (n = 25) could be distinguished from those of control subjects (n = 6) based solely upon the expression of these 231 “albumin-regulated genes.” The expression of an 11-transcript subset related to the degree of proteinuria, and this 11-mRNA subset was also sufficient to distinguish biopsies of subjects with IgAN from control biopsies. We tested if these findings could be extrapolated to other proteinuric diseases beyond IgAN and found that all forms of primary glomerulonephritis (n = 33) can be distinguished from controls (n = 21) based solely on the expression levels of these 11 genes derived from our *in vitro* proteinuria model. Pathway analysis suggests common regulatory elements shared by these 11 transcripts. In conclusion, we have identified an albumin-regulated 11-gene signature shared between all forms of primary glomerulonephritis. Our findings support the hypothesis that albuminuria may directly promote injury in the tubulo-interstitial compartment of the kidney in patients with glomerulonephritis.

## Introduction

Proteinuria is the clinical hallmark of glomerulonephritis, and the most important predictor of outcome in both diabetes-related and idiopathic glomerular-based kidney disease [1]–[8]. IgA nephropathy (IgAN) is the most common form of primary kidney disease world-wide [9], [10]; up to 40% of patients with IgAN progress to renal failure within 10 years of diagnosis [11]. Studies have consistently shown that proteinuria is the most powerful predictor of the rate of kidney function decline and kidney survival in IgAN [12]–[17], and that in patients with IgAN, this relationship is particularly strong even at low levels of proteinuria [18].

One of the pathologic features common to all forms of progressive glomerular-based kidney disease is tubulo-interstitial fibrosis, which shows consistent correlation with renal functional impairment [19]–[21]. Tubulo-interstitial fibrosis may be triggered by a variety of processes [22]; one proposed mechanism includes exposure of tubular cells to protein. Experimental evidence suggests that proteinuria is not only a marker of disease progression, but is directly involved in the pathogenesis of tubulo-interstitial fibrosis, and the progression of kidney injury [23], [24]. In patients with glomerular disease, proximal tubular epithelial cells are exposed to pathologically high concentrations of urinary proteins, including albumin. This induces a number of potentially injurious biologic responses in tubular epithelial cells, including inflammation, apoptosis, production of reactive oxygen species, and transition to a myofibroblast phenotype, ultimately contributing to tubulo-interstitial fibrosis [25]–[30]. These cellular responses may be dependent upon direct receptor-mediated uptake of albumin by tubular epithelial cells and subsequent stimulation of down-stream responses (such as NFκB-dependent gene transcription) or endocytosis-independent activation of signaling cascades by albumin [31]. While tubular cell exposure to protein is not the only proposed mechanism by which glomerular diseases result in tubulo-interstitial injury and progressive loss of renal function [22], [32], these observations may explain, in part, the important relationship between proteinuria, tubulo-interstitial injury, and long term outcome in glomerular-based kidney disease [19]–[21], [33].

Genome wide mRNA expression profiling tools, combined with robust statistical approaches, provide an unbiased approach to study the tubulo-interstitial transcriptional response initiated by proteinuria [34]. Using this strategy, we have demonstrated in an *in vitro* model of proteinuria that exposure of primary human renal proximal tubular epithelial cells to albumin induces the differential mRNA expression of a number of “albumin-regulated” genes, including interleukin-8 (IL-8) and the epidermal growth factor receptor (EGFR) [29]. Using this model system we demonstrated that albumin exposure *in vitro* results in the enhanced expression of IL-8 via activation of the mitogen-associated protein kinase ERK, an effect that was dependent upon transactivation of the EGF receptor and the generation of reactive oxygen species. While this *in vitro* model is a highly simplified representation of the *in vivo* disease process, *in vitro* findings using similar systems have been confirmed in studies of human kidney disease [30], [35].

To better understand the relationship between proteinuria and tubular epithelial cell responses, we studied the expression of “albumin-regulated genes”, defined in vitro, in the tubulo-interstitium of human kidney biopsies from patients with glomerulonephritis. First, primary human renal tubular epithelial cells were exposed to albumin *in vitro*, and differential gene expression was assessed using mRNA microarray analysis. A set of 231 differentially expressed “albumin-regulated” genes was derived, and the expression of these genes was then measured in the tubulo-interstitial compartment of kidney biopsy tissue from patients with primary glomerulonephritis and healthy live kidney donors. We first studied the expression of these transcripts in IgAN biopsies, given that this is the most common type of primary glomerulonephritis, and given the particularly close relationship between proteinuria and kidney function in this disease [18]. We then studied mRNA expression in the tubulo-interstitial compartment of patients with other forms of idiopathic glomerulonephritis to determine if there are shared mechanisms of tubulo-interstitial injury.

## Results

### Differential gene expression in an *in vitro* model of proteinuria

To determine the effect of albumin on gene expression in cultured primary human renal tubular epithelial cells, mRNA expression was measured using data from 8 microarrays (4 with control conditions representing 8 experimental *in vitro* replicates, and 4 BSA-treated conditions, representing 8 experimental replicates). Using conservative thresholds for differential gene expression, we identified 231 transcripts differentially expressed in cells treated with 1% bovine serum albumin (BSA) versus control conditions for 6 hours. A selection of the mRNA transcripts found to be differentially expressed in this model, using stringent statistical selection criteria, is provided in Table 1 (full list Supplementary Data S1). Albumin-dependent mRNA regulation was seen in genes involved in apoptosis, cell growth and metabolism, cell-signaling, lipid metabolism, matrix turnover, cell cycle, cell movement, lipid metabolism, and reactive oxygen species scavenging.

**Table 1:** Genes differentially expressed in cells exposed to albumin.

**Genes related to apoptosis: p = 0.02**		**Genes related to cell cycle: p = 0.02 CONT'D**	
BIN1	bridging integrator 1	INHBA	inhibin, beta A (activin A, activin AB alpha polypeptide)
CDK2AP1	CDK2-associated protein 1	KPNA2	karyopherin alpha 2 (RAG cohort 1, importin alpha 1)
CDKN1A	cyclin-dependent kinase inhibitor 1A (p21, Cip1)	MSH2	mutS homolog 2, colon cancer, nonpolyposis type 1 (E. coli)
CFLAR	CASP8 and FADD-like apoptosis regulator	PTHLH	parathyroid hormone-like hormone TFDP1 transcription factor Dp-1
CROP	cisplatin resistance-associated overexpressed protein	TFDP2	transcription factor Dp-2 (E2F dimerization partner 2)
DAPK1	death-associated protein kinase 1	TYMS	thymidylate synthetase
DUSP5,6	dual specificity phosphatase 5,6		
GLRX	glutaredoxin (thioltransferase)	**Cellular movement: p = 0.02**	
HTRA2	HtrA serine peptidase 2	HMGB2	high-mobility group box 2
IER2	immediate early response 2	NR2F2	nuclear receptor subfamily 2, group F, member 2
IER3	immediate early response 3	RGS4	regulator of G-protein signaling 4
IGFBP3	insulin-like growth factor binding protein 3	S100A2	S100 calcium binding protein A2
MBD4	methyl-CpG binding domain protein 4	SPRY2	sprouty homolog 2 (Drosophila)
MCL1	myeloid cell leukemia sequence 1 (BCL2-related)	SPRY4	sprouty homolog 4 (Drosophila)
NDRG1	N-myc downstream regulated gene 1		
SFN	stratifin	**Connective tissue development and metabolism: p = 0.02**	
THBD	thrombomodulin	COL1A1	collagen, type I, alpha 1
		CYR61	cysteine-rich, angiogenic inducer, 61
**Cell growth and proliferation: p = 0.03**		KRT18	keratin 18
ADFP	adipose differentiation-related protein	PLAU	plasminogen activator, urokinase
CCL20	chemokine (C-C motif) ligand 20	PLAUR	plasminogen activator, urokinase receptor
DNAJC9	DnaJ (Hsp40) homolog, subfamily C, member 9	SERPINE1	serpin peptidase inhibitor, clade E (nexin, plasminogen activator inhibitor type 1), member 1
HSPB8	heat shock 22kDa protein 8		
IRF8	interferon regulatory factor 8	**Lipid metabolism: p = 0.02**	
SEMA4D	sema domain, immunoglobulin domain (Ig), transmembrane domain (TM) and short cytoplasmic domain, (semaphorin) 4D	ACSL3	acyl-CoA synthetase long-chain family member 3
UPP1	uridine phosphorylase 1	FDFT1	farnesyl-diphosphate farnesyltransferase 1
		HMGCR	3-hydroxy-3-methylglutaryl-Coenzyme A reductase
**Cell-to-cell signaling, interaction: p = 0.003**		HMGCS1	3-hydroxy-3-methylglutaryl-Coenzyme A synthase 1 (soluble)
EGFR	epidermal growth factor receptor (erythroblastic leukemia viral v-erb-b oncogene homolog, avian)	LDLR	low density lipoprotein receptor (familial hypercholesterolemia)
EGR1	early growth response 1	LIPG	lipase, endothelial
ELF3	E74-like factor 3 (ets domain transcription factor, epithelial-specific)		
IGFBP3	insulin-like growth factor binding protein 3	**Free radical scavenging: p = 0.01**	
IL6	interleukin 6 (interferon, beta 2)	TXNIP	thioredoxin interacting protein
IL8	interleukin 8	XDH	xanthine dehydrogenase
LIF	leukemia inhibitory factor (cholinergic differentiation factor)		
PBEF1	pre-B-cell colony enhancing factor 1	**Other Genes of Interest**	
PHLDA2	pleckstrin homology-like domain, family A, member 2	BHLHB2	basic helix-loop-helid domain containing, class B,2
TNFSF10	tumor necrosis factor superfamily, member 10	CEBPD	CCAT/enhancer binding protein, delta
		KDELC1	KDEL (Lys-Asp-Glu-Leu) containing 1
**Genes related to cell cycle: p = 0.02**		MAFF	v-maf musculoaponeurotic fibrosarcoma oncogene homolog F (avian)
CDC7	CDC7 cell division cycle 7 (S. cerevisiae)	PSMD11	proteasome (prosome, macropain) 26S subunit, non-ATPase, 11
CDC27	cell division cycle 27	SAMD4	sterile alpha motif domain containing 4
CDK4	cyclin-dependent kinase 4	SLC19A2	solute carrier family 19 (thiamine transporter) member 2
G0S2	G0/G1switch 2	STEAP1	6 transmembrane epithelial antigen of prostate 1
HBEGF	heparin-binding EGF-like growth factor	TARS	threonyl-tRNA synthetase
HK2	hexokinase 2	ZBED5	zinc finger BED-type containing 5
HMOX1	heme oxygenase (decycling) 1		

### Expression of “albumin-regulated genes” in human kidney biopsy sample

Affymetrix microarray mRNA expression data were generated from the tubulo-interstitial compartment of biopsies from 25 patients with IgAN and proteinuria and 6 control subjects enrolled in the European Renal cDNA Bank Bank - Kröner-Fresenius biopsy bank (ERCB-KFB). The clinical characteristics of subjects are shown in Table 2; subjects with IgAN had a wide range in proteinuria (trace to 10g/day) and renal function (normal to stage 4/5 CKD). Expression data for 231 unique “albumin-regulated” genes (derived above) were extracted from the human renal biopsy microarray data.

**Table 2:** Patient characteristics – subjects with IgA nephropathy.

	Control	IgA nephropathy
Number	6	25
Mean age(range)	48.5(32–62)	36(19–84)
% male	50	71
Mean serum creatinine ± SD (range)µmol/L	<120	131±129 (40–643)
Mean 24h urine protein ± SD (range)g/24h	<0.20	2.36±2.3 (0.28–10)

**Figure 1:**
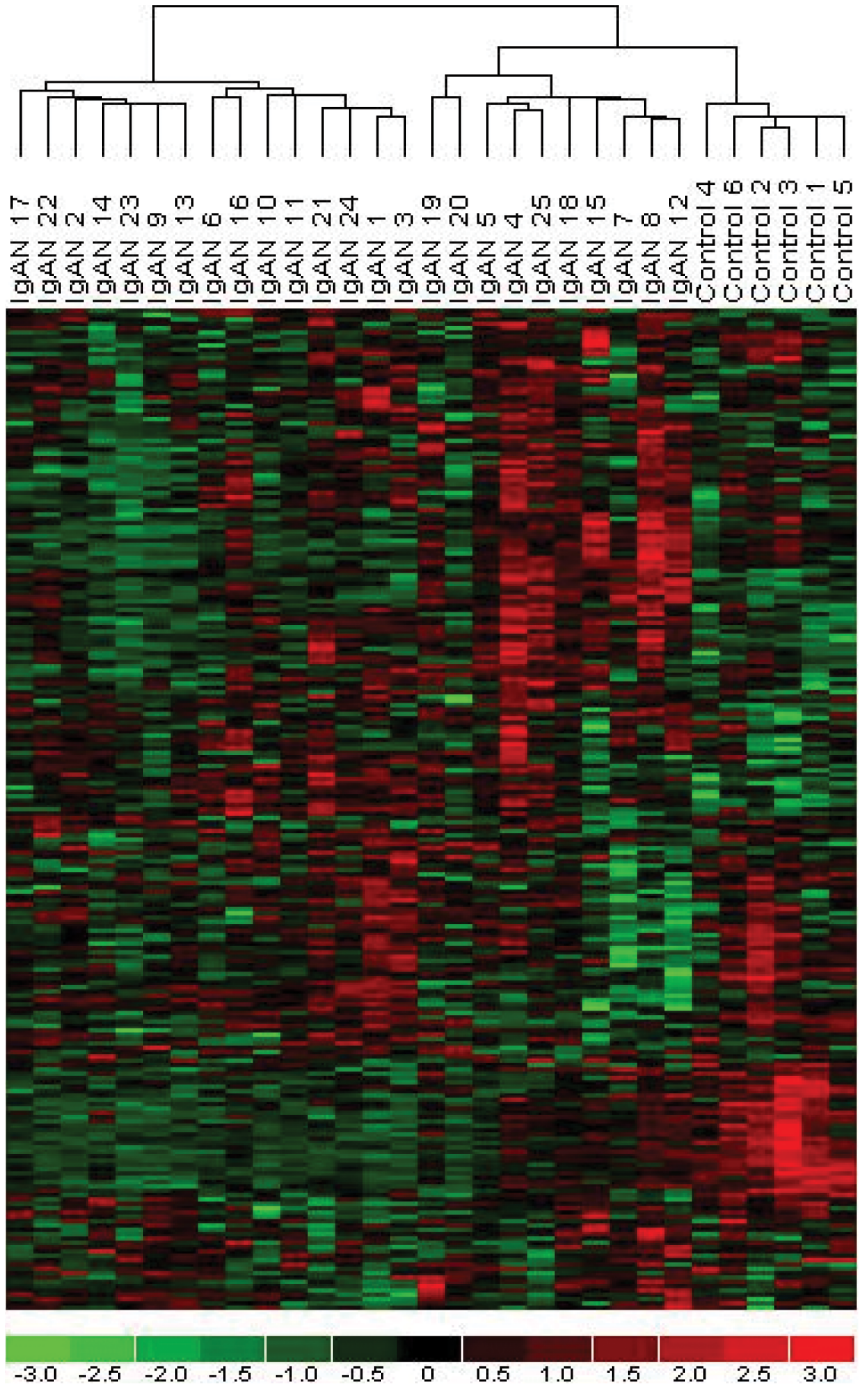
Cluster analysis. Clustering of microarrays according to disease status (horizontal axis) based upon expression of the 231 albumin-regulated genes (vertical axis). Control = control group, IgAN = IgA nephropathy.

To determine if the expression levels of these 231 genes were associated with the kidney disease process, hierarchical cluster analysis of the expression data was performed (Figure 1). Cluster analysis distinguished between renal biopsies from control subjects and the renal biopsies from patients with IgAN based upon the expression of the 231 “albumin-regulated genes”. The cluster analysis findings were not reproduced using the expression data of randomly selected gene sets of similar size. Of the 231 genes, 49 (21%) were differentially expressed in the IgAN samples compared to the healthy control samples (FDR 5%, see Supplementary Data S1) compared to 4% of the genes in the complete Affymetrix data (χ^2^ = 147, p<0.01), confirming the enrichment of regulated genes in the *in vitro* defined gene set.

### A gene expression signature of proteinuria in IgAN

Given the critical relationship between proteinuria and outcome in IgAN, even at low levels of proteinuria [18], we specifically examined genes differentially expressed in IgAN biopsies compared with control biopsies. The 231 gene set was derived from an *in vitro* model of proteinuria designed to be representative of the biologic response of kidney tubular cells in to proteinuria, so we studied the relationship between the expression levels of the “albumin-regulated” genes in the tubulo-interstitial compartment from the renal biopsies of the patients with IgAN with the level of proteinuria at the time of kidney biopsy, and selected the mRNAs with expression levels significantly correlated with proteinuria.

We then utilized the following criteria to select a list of genes for the validation studies: 1) genes differentially expressed by human renal tubular cells after exposure to albumin, *in vitro*; 2) genes differentially expressed in the tubulo-interstitial compartment of patients with IgAN compared to control subjects (this compartment includes tubular epithelial cells, interstitial tissue and cells such as fibroblasts, and endothelial cells in vascular structures) based upon the biopsy Affymetrix microarray data set; 3) genes with expression levels related to level of proteinuria within IgAN. This selection process yielded a set of 11 genes (see Table 3).

**Table 3:** Validation gene set.

Gene Title	Gene Symbol	Biological Process Description
collagen, type I, alpha 1	COL1A1	skeletal development, epidermis development
early growth response 1	EGR1	regulation of transcription, T cell differentiation
E74-like factor 3 (ets domain transcription factor,epithelial-specific)	ELF3	regulation of transcription, epidermis development, morphogenesis
immediate early response 3	IER3	Anti-/apoptosis, morphogenesis
heparin-binding EGF-like growth factor	HBEGF	Signal transduction, EGFR signaling pathway, smooth muscle cell proliferation
v-maf musculoaponeurotic fibrosarcoma oncogene homolog F (avian)	MAFF	transcription regulation, epidermal cell differentiation
myeloid cell leukemia sequence 1 (BCL2-related)	MCL1	regulation of apoptosis, differentiation
sterile alpha motif domain containing 4A	SAMD4A	positive regulation of translation
serpin peptidase inhibitor (plasminogen activator inhibitor type 1)1	SERPINE1	blood coagulation, fibrinolysis, regulation of angiogenesis
six transmembrane epithelial antigen of the prostate 1	STEAP1	electron transport
Thymidylate synthetase	TYMS	nucleic acid metabolism/biosynthesis, DNA replication/repair, phosphoinositide-mediated signaling

### Albumin-regulated genes in primary GN

In order to determine if the relationship between the expression of the albumin-regulated genes and the kidney disease process is unique to IgAN, the Affymetrix microarray mRNA expression data for these genes were derived from the tubulo-interstitial compartment of biopsies from patients with primary focal segmental glomerulosclerosis (FSGS, n = 10), membranous GN (MGN, n = 18) and minimal change disease (MCD, n = 5) and proteinuria and 21 control subjects (healthy kidney donors with normal renal biopsies) enrolled in the ERCB-KFB. The clinical characteristics of subjects are shown in Table 4. As reference samples healthy living kidney donors known to have normal renal function and no proteinuria were used.

**Table 4:** Patient characteristics – European Renal cDNA Bank glomerular disease subjects.

	FSGS	MGN	MCD
Number	10*	18	5**
Mean age (range)	50 (32–76)	53 (18–86)	45 (25–78)
% male	60	56	80
Mean serum creatinine ± SD (range)µmol/L	156±105 (53–355)	88.6±35.5 (40–160)	101±42.5 (53–134)**
Mean 24h urine protein ± SD (range)g/24h	4.97±2.6 (1.9–8.4)	4.58±3.2 (0.5–9.8)	2.90±4.0 (0.1–5.7)

*10 patients age available for only 4 patients.

**5 patients; age and serum creatinine available for only 3 patients.

**Figure 2:**
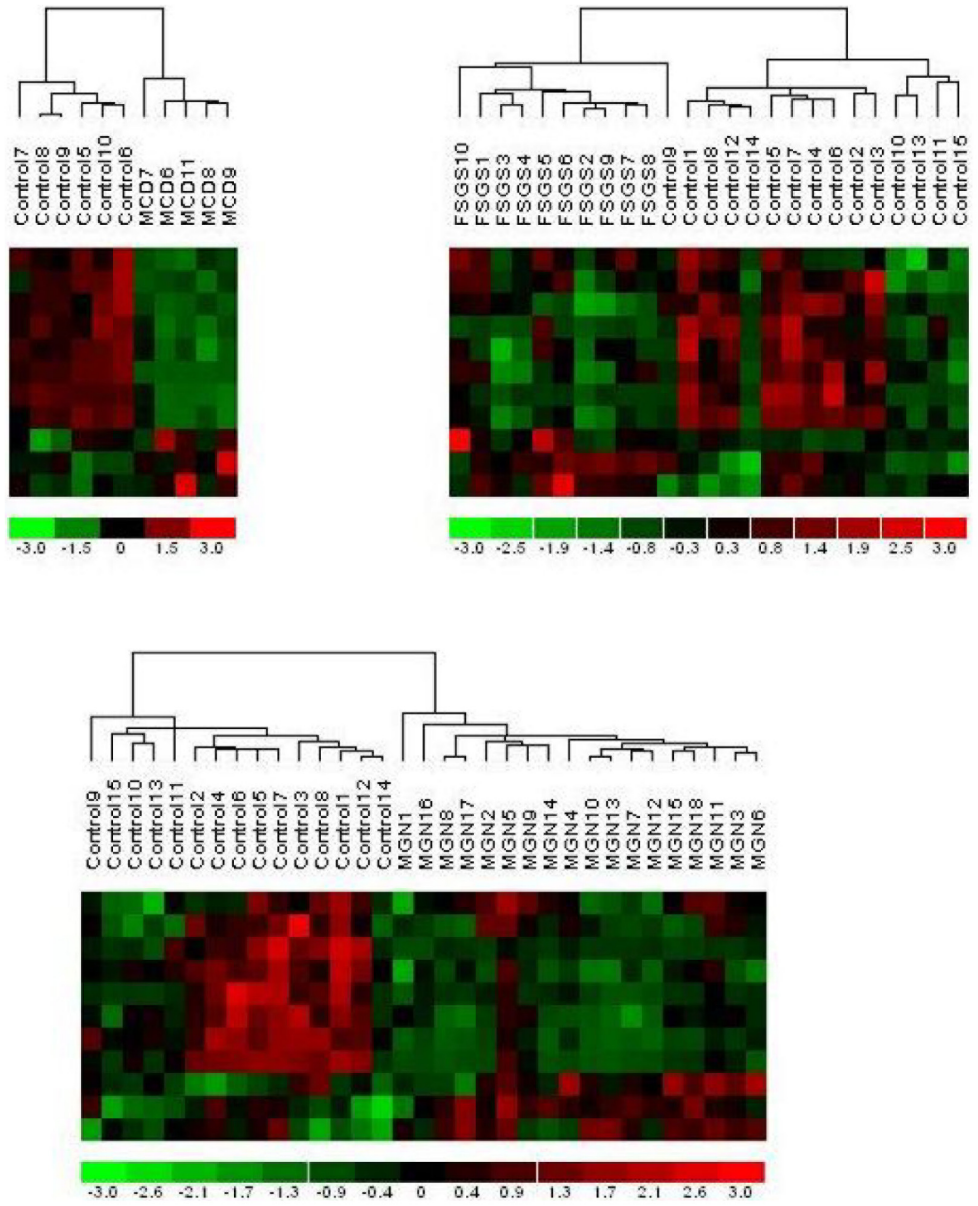
Cluster analysis. Clustering of microarrays according to disease status (columns) based upon expression of the 11 albumin-regulated genes (rows). Control = control group, FSGS = focal segmental glomerulosclerosis, MGN = membranous GN, MCD = minimal change disease.

We found that the expression of these 11 mRNAs differed in the tubulo-interstitial compartment of all biopsies of subjects with primary glomerular disease compared to controls. Hierarchical cluster analysis confirmed that renal biopsies from healthy donors can be distinguished from the renal biopsies of patients with all forms of primary GN based solely upon the expression of the 11 mRNA gene signature (Figure 2). Cluster analysis using expression data of randomly selected gene sets of similar size did not distinguish kidney biopsies of control subjects from the biopsies of subjects with GN.

### Potential common regulatory pathways

**Figure 3:**
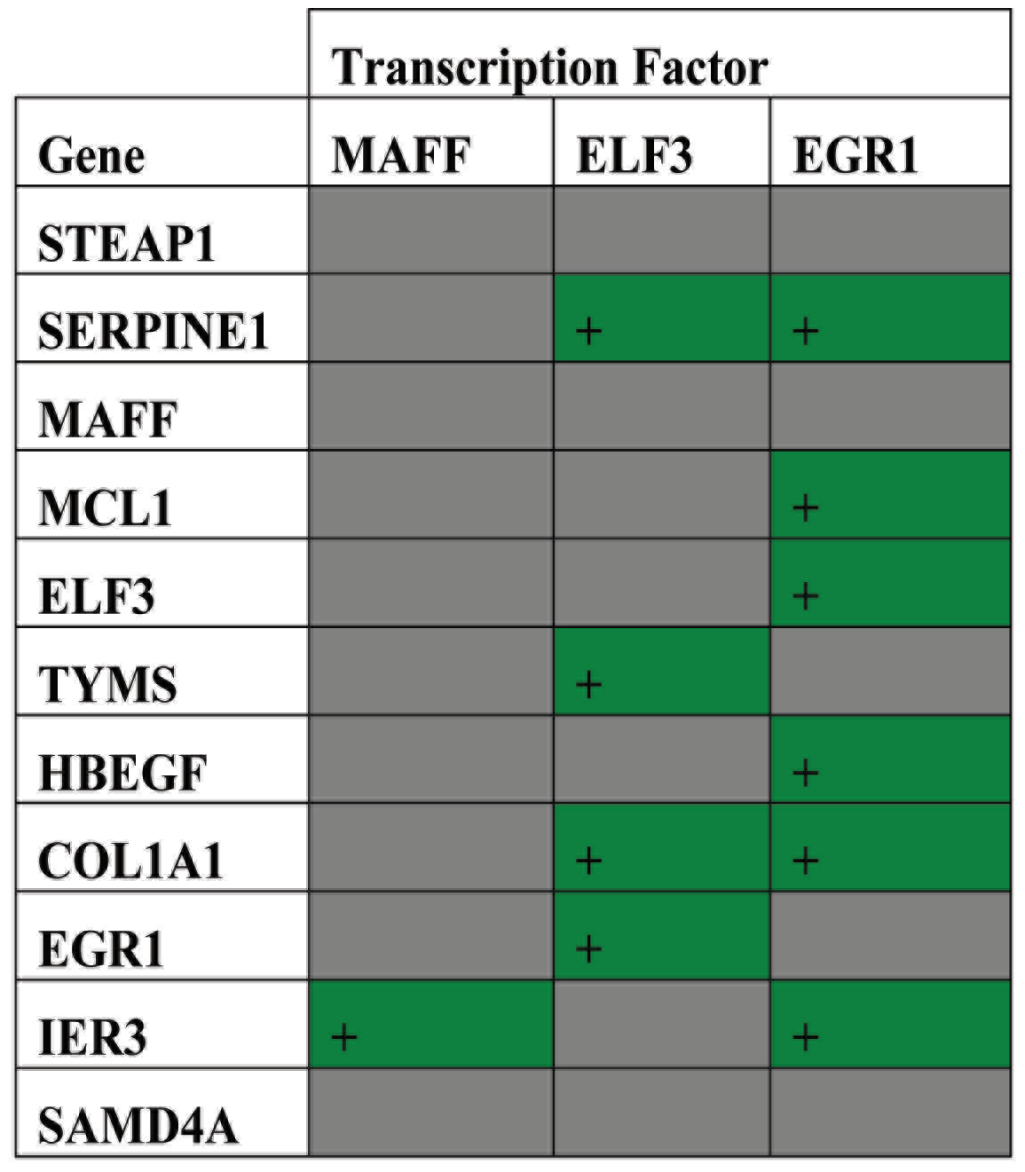
Transcription factor analysis. Green shade indicates the presence of a transcription factor binding site sequence, upstream from the gene of interest. Each row represents a gene of interest, and each column represents a putative transcription factor.

To define the functional context of the proteinuria associated genes, a transcriptional network was constructed using a co-citation natural language processing (NLP) tools (Genomatix Bibliosphere), considering all of the transcripts of the 11-gene mRNA signature (Supplementary Data S1). EGR1 emerged as a central node linking EGR1 to the remaining 10 mRNA transcripts. To determine if common transcription factor promoter elements could explain the functional relationship between these 11 albumin-regulated mRNA transcripts, promoter regions (500bp up and 100bp downstream of the transcription start sites) was performed (Genomatix Bibliosphere). In concordance with the central role of the EGR node in the NLP analysis, most of the genes encoding the 11-gene mRNA signature contain a proximal EGR1 promoter regions, consistent with a putative common transcriptional regulation (Figure 3).

## Discussion

Proteinuria is an important determinant of outcome in primary GN but the mechanisms responsible for this association have not been fully elucidated. Although *in vitro* and experimental studies suggest that proteinuria, and in particular albumin, elicits a biological response in kidney tubule epithelial cells that contributes to progressive tubulointerstitial injury [35], it is uncertain if proteinuria has a direct effect on gene expression in human kidney disease. Development of new unbiased molecular and statistical tools for studying mRNA expression in renal tissue has greatly advanced our ability to study renal disease, and to translate findings from basic molecular and cell biology research to human disease [34], [36]–[44]. Accordingly, the aim of this study was to test the hypothesis that there is a steady-state change in gene expression in the renal tubulointerstitium of subjects with primary GN that reflects a biological response of the tubule cells to proteinuria.

In order to address this hypothesis we exposed primary human renal proximal tubular epithelial cells to albumin, *in vitro*, simulating the exposure of tubular cells to proteinuria in human proteinuric glomerulonephritis. Our first major finding was the identification of a distinct set of 231 mRNAs differentially regulated in human renal tubular cells by albumin exposure *in vitro* (Supplementary Data S1). Gene ontology (GO) analysis identified several pathways that were statistically over-represented in the *in vitro* expression data, and the proteins encoded by these genes are involved in diverse biological processes including apoptosis, cell cycling, pro-inflammatory cell signaling cytokines, connective tissue development and fibrosis, and free radical scavenging, and lipid metabolism (Table 1).

Although the genes regulated by albumin *in vitro* can be related to injury pathways and outcomes like cell loss, inflammation, and fibrosis, we sought to translate these findings to human kidney disease by comparing the expression levels of these genes in the kidneys of normal subjects and subject with primary GN. mRNA levels were measured by microarray analysis in the microdissected tubulointerstitial compartment of renal biopsy samples in order to capture the kidney tubular cell transcriptome *in vivo*. We chose to focus first on subjects with IgAN because new evidence has shown that incremental increases in proteinuria are associated with dramatic reductions in renal survival in IgAN [18], and that in patients with IgAN, these changes occur at far lower levels of proteinuria compared to patients with other forms of primary GN [45]. Our second major finding was that we could distinguish between the kidney biopsies of subjects with IgAN and healthy living donors based solely upon the tubulo-interstitial expression levels of the 231 “albumin-regulated genes” using hierarchical cluster analysis.

In order to determine if this clustering phenomenon was a chance event, we tested random sets of 231 genes generated from the full Affymetrix expression dataset but cluster analysis of these random gene sets failed to segregate IgAN from control biopsies. In addition, we found significantly enriched differential expression of the 231 “albumin-regulated genes” in the tubulo-interstitial tissue of IgAN biopsies compared to control biopsies. Taken together, these analyses support the hypothesis that the biological response to albumin that we observed *in vitro* may also be present, at least in part, in the kidneys of subjects with IgAN and proteinuria.

In order to further explore the link between proteinuria and gene expression *in vivo*, we then studied the relationship between the expression levels of the 231 genes and the levels of proteinuria at the time of biopsy within the group of subjects with IgAN. The rationale for this analysis was twofold: first, proteinuria is known to be the most powerful predictor of outcome in glomerular-based diseases, including IgAN, and, second, the tubular response to proteinuria (modeled by our *in vitro* experiment) may be a key factor determining progressive tubulo-interstitial fibrosis and nephron loss, important pathologic indicators of prognosis in IgAN [11], [18]. Our third major finding was that the tubulo-interstitial expression levels of 11 mRNA transcripts (of the 231 albumin-regulated genes) correlated significantly with the level of proteinuria at the time of biopsy, suggesting that there may be a biological relationship between gene expression in the tubulointerstitial compartment of the kidney and proteinuria. We labeled this set of 11 genes the “proteinuria signature”.

The 11 genes in the “proteinuria signature” encode some proteins previously implicated in the tubular response to proteinuria, as well as some proteins that have not been studied in the context of kidney disease, and thus may be potential new mediators of progressive kidney injury. For example, protein members of the coagulation cascade have been implicated in extracellular matrix protein turnover in the kidney, and we found that Serpine1 (PAI-1) was a transcipt identified in the proteinuria signature. Serpine1 is not normally produced in the kidneys, however experimental evidence suggests that it is an important promoter of renal fibrosis [46]. The mechanisms by which it promotes fibrosis are not entirely elucidated, however in addition to inhibiting protease activity in the extracellular compartment, it may modulate inflammatory cell recruitment, and fibroblast activation [47]. Work in experimental models of kidney disease including protein overload injury, proliferative glomerulonephritis, and obstructive kidney disease suggests that increased PAI-1 expression is associated with interstitial fibrosis, and reduction in PAI-1 expression (via drug therapy or recombinant techniques) is associated with attenuation of renal fibrotic injury [46]–[50]. Furthermore, de novo PAI-1 protein expression is documented in kidney biopsies of patients with glomerular based kidney disease [51]–[53]. Our data support the hypothesis that proteinuria may contribute to fibrosis by increasing PAI-1 expression by tubular cells. In addition, the early growth response gene 1 (EGR-1) has been implicated in the TGFβ-mediated fibrosis [54] and in the regulation of interstitial fibrosis in the experimental unilateral ureteric obstruction model [55]. Finally, our discovery that the gene for collagen I, alpha 1, was also in the “proteinuria signature” also supports a link between proteinuria, gene expression in the tubulointerstitium, and fibrosis.

In addition to interstitial fibrosis, apoptosis of tubular cells contributes to progressive kidney injury in primary GN by promoting cell loss [56], [57]. In this regard, EGR-1 has also been reported to regulate apoptosis in response to oxidative stress [58]. Two other apoptosis-related genes, immediate early response 3 (IER3) and myeloid cell leukemia sequence 1 (MCL1), were also in the 11 gene “proteinuria signature”. The MCL1 gene encodes a member of the bcl2 family that may promote or inhibit apoptosis depending on the tissue [59]–[61]. For example, MCL1 has been found to support cell survival in Wilms' Tumour cell lines [62]. Neither MCL1 nor IER3 have been studied in the context of primary GN, and further studies will be necessary to better define their role in kidney disease. Similarly, the role of the proteins encoded by genes MAFF, TYMS, and SAMD4A in the progression of GN is unknown.

The next goal of this study was to determine if these 11 genes represented a generic response to proteinuria that was present in other forms of primary GN because our *in vitro* experiments identified a response of tubular cells to albumin that was independent of glomerular injury. In order to address this question, we studied the expression levels of the 11 genes in kidney biopsy samples from subjects with three other common forms of primary GN: focal segmental glomerulosclerosis (FSGS), membranous nephropathy (MGN), and minimal change disease (MCD). The biopsies of subjects with FSGS, MGN, and MCD could be distinguished from control biopsies based solely upon the expression of the 11-gene signature, supporting the conclusion that this 11-gene set is part of a common pathway linking proteinuria to gene expression in the kidney.

Finally, we subjected the 11 genes to a bioinformatics analysis in order to explore relationships between the component genes in the “proteinuria signature”. We first constructed a transcriptional network (Supplemental Figure S1 in Supplementary Data S1) utilizing Genomatix Bibliosphere. The network derived from this analysis placed EGR1 in the central node. Based on this finding, we then went on to perform a transcription factor analysis utilizing gene promoter sequence data for the 11 genes, and we found that the consensus sequence for EGR1 was present in 6 of the genes in the “proteinuria signature”. This analysis suggests that EGR1 may play a key role in a common pathway orchestrating the transcriptional response kidney tubule cells to proteinuria *in vivo*. The transcription factor analysis further suggests that the transcription factor ELF3 may also mediate this response, at least in part.

There are some important limitations in the current study. First, stringent and conservative statistical thresholds were used in the initial selection of differentially expressed genes *in vitro* at a single time point of 6-hours of albumin exposure. This design may have precluded identification of other important genes involved in the tubular cell response to albumin exposure (Type II Error). In addition, we used the in vitro data set for a targeted analysis of the human subjects with GN, capturing a different gene set than that identified by Rudnicki et al. [63] in studies of laser-microdissected proximal tubular cells in GN.

Finally, while we derived our list of candidate genes from an *in vitro* model of proteinuria, it is likely that the transcriptional response of the genes measured in the tubulo-interstitium of the kidney biopsies is not due entirely to exposure to albumin the ultrafiltrate. We may also be capturing the tubular response to other factors that play a role in progression. For example, it is possible that the transcriptional response is related to exposure of tubular cells to filtered growth factors [64], other proteins in the ultrafiltrate, or modified albumin moieties [42], [65]–[69]}. In this regard, Kritz and coworkers have suggested that albumin uptake by tubular cells is not an absolute prerequisite for tubulo-intertitial injury in mice with glomerulonephritis [32]. Other contributors to nephron loss in glomerular-based kidney disease include misdirected filtrate and obstruction of the tubulo-glomerular junction with subsequent tubular cell injury and ischemia [22], [32]. The importance of multifactorial contributions to tubulo-interstitial fibrosis beyond tubular cell albumin exposure is highlighted by the clinical observation that in IgAN, tubulo-interstitial injury and functional decline occurs at far lower levels of proteinuria [45]. Of interest, our mRNA signature was able to discriminate minimal change nephropathy biopsies from control samples, despite the fact that this entity is only more rarely associated with tubulo-interstitial fibrosis and progressive functional loss [70]. However, when proteinuria reduction is not achieved in patients with this disease, sclerosis, fibrosis, and functional loss does occur [70].

It is also possible that the tissue mRNA signature is not entirely derived from tubular cells. There are little morphometric data that quantify the relative abundance of cells that comprise the cortical interstitium in the disease state. In “normal” control biopsies, 87% of the tubulo-interstitial compartment is comprised of the tubular cell component; in biopsies of patients with type 1 diabetes with early nephropathy, this reaches 91% [71]. The non-tubular cell component comprises only 13% of the healthy cortex or less [72]. Of the non-tubular cell portion, only 14% (ie. <2% total) is composed of peritubular capillaries, in biopsies with moderate interstitial expansion due to diabetic nephropathy [71], [72]. Taken together, these data suggest that the predominant resident cell transcriptome signal is derived from tubular cell mRNA expression. Emerging evidence also suggests that number of dendritic cells in the cortical tubulo-interstitium of kidney biopsies from patients with glomerulonephritis is increased in comparison to control biopsies [73], and appear to contribute to progressive kidney disease in animal models [74]. Interstitial macrophage infiltration occurs in many forms of primary glomerulonephritis; while data suggest that cellular infiltration correlates with renal function at the time of biopsy, the relationship to proteinuria is not as clear (reviewed in [75]). We cannot discount the possibility that these cells are also contributing to the mRNA expression profile.

In summary, we have used an *in vitro* model of proteinuria to identify a set of “albumin-regulated genes” in primary human renal tubular cells. We have translated these findings to human primary GN, and identified a subset of mRNA transcripts with expression levels that correlate with the level of proteinuria, and that distinguish biopsies of subjects with GN from biopsies of control subjects. Further studies will be necessary to define the biological role of these genes in proteinuric kidney disease and to determine if measures of expression of these genes are predictive of long-term clinical outcome.

## Methods

### Primary cell culture system

The cell system used was previously described [29]. Eight flasks of primary human renal tubular epithelial cells (Cambrex, Walkersville, MD) were exposed to medium alone and 8 flasks of cells were exposed to medium containing 1% bovine serum albumin for 6 h. The RNA extracted (Qiagen RNeasy kit, Valencia, CA); RNA from cells grown in two flasks was pooled to form one experimental sample, and each experiment was performed in quadruplicate (total 16 flasks for eight microarrays—four arrays from control cell RNA and four arrays from albumin-treated cell RNA). RNA quality was verified using the Agilent bioanalyzer (Agilent Technologies, Palo Alto, CA).

Synthesis of cDNA and array hybridization, washing, and scanning were performed by the Affymetrix Gene Chip core facility at The Centre for Applied Genomics at Toronto's Hospital for Sick Children (Ontario, Canada) according to Affymetrix-recommended protocols (Santa Clara, CA) using the hgu 133A Affymetrix Gene Chip and an Affymetrix Fluidics station.

### RNA extraction and mRNA expression profiling of human renal biopsy tissue

The study was performed as outlined previously in detail [36], [40]. Human renal biopsy specimens were procured in an international multicenter study, the European Renal cDNA Bank- Kröner-Fresenius biopsy bank (ERCB-KFB, see acknowledgements for participating centers) [36]. Renal biopsies were obtained after written consent and approval of the ethics committee and in the frame of the ERCB-KFB approved by the specialized subcommittee for internal medicine of the cantonal ethics committee of Zurich. The characteristics of patients are shown in Table 2. Control biopsy samples were obtained during the cold ischemia period of living-related donor transplantation.

Total RNA was extracted from manually microdissected tubulo-interstitial compartments obtained from living donors (n = 6) and patients with IgA nephropathy (n = 25). After one round of amplification of 300–800 ng of total RNA, RNA quality and quantity was verified (Agilent Technologies, Waldbronn, Germany). The fragmentation, hybridization, staining and imaging was performed according the manufacturer's guidelines (Affymetrix). For a detailed description of the protocol see reference [44]. All microarray data are MIAME compliant as detailed on the MGED Society website http://www.mged.org/Workgroups/MIAME/miame.html. The raw data will be GEO accessible through GEO Series accession.

### II. Data filtering strategy to determine renal response to proteinuria

In order to rationally filter the large volume of data derived from the microarray experiments, the following strategy was employed to select the genes that are characteristic of the renal response to proteinuria:

- 1 – Identification of genes differentially expressed in the *in vitro* model of proteinuria by SAM and Limma analysis (described below).
- 2 – Identification of genes differentially expressed in the mRNA expression profiling data from tubulo-interstitial tissue of patients with IgA nephropathy vs. control samples.
- 3 – Identification of genes correlating with and predictive of proteinuria *in vivo* by linear models using Limma and lasso regression procedure, respectively.

### Statistical tools employed for data analysis

The microarray data obtained from the *in vitro* model were examined and visualized using Affymetrix Microarray Suite 5.0 software and Bioconductor [76], [77]. The calculation of expression values from probe intensities and normalization of arrays was performed using the RMA method [78] using Bioconductor and RMAexpress [79] (accessed 2006). Differential gene expression was determined using Limma (Linear models for microarray data) and SAM (Significance Analysis of Micoarrays) through Bioconductor [80], [81], with a highly conservative false-discovery rate set at 0.01, and genes were not filtered based upon an arbitrarily-selected fold-change in expression. Differential expression was assessed in the *in vivo* tubulo-interstitial samples using SAM and dChip [82].

Cluster analysis was performed using Sammon mapping/multi-dimensional scaling, as well as spectral clustering [83] for experimental cell data, and hierarchical cluster analysis was performed using dChip [82], [84] in the renal biopsy dataset (centroid-based, distance metric: 1-correlation).

In order to explore the ontology of genes differentially expressed in vitro, genes were ranked by limma topTable function (by adjusted p-value), and 600 up and down-regulated genes were selected to study possible common ontology patterns. Enriched expression of gene ontology (GO) terms was assessed with Ingenuity Pathway Analysis Software 4.2 (Redwood City, CA) and confirmed using the Bioconductor package GOstats. These programs determine which gene ontology terms found in gene lists are statistically over or under represented, compared with the GO terms represented in the microarray as a whole [85], [86]. A list of enriched GO terms is produced, including the test statistic and associated p value, suggesting functional mechanisms that may underlie the biological response captured in the data set.

Clinical data were extracted for the patients who underwent renal biopsy and inspection revealed that proteinuria values and residuals were skewed, and should be normalized by log transformation for regression analysis. To select transcripts with mRNA expression most closely related to proteinuria in IgAN, mRNA expression was correlated with proteinuria *in vivo* using advanced regression analysis with linear models (with limma and topTable function in Bioconductor) [81]. Partitioning methods were also employed to use the biopsy gene expression data to predict proteinuria. Lasso regression procedure was also used to confirm genes that were most predictive of log proteinuria tuned by a 10-fold cross-validation procedure [87].

Once this filtration strategy was applied, and the 11-mRNA signature identified, the normalized mRNA expression data were then extracted from the full datasets from MGN, FSGS, and MCD biopsies. Hierarchical cluster analysis was performed on the human renal biopsy data set using dChip [82] (centroid-based, distance metric: 1-correlation). Tests of the correlation between proteinuria and mRNA expression were performed by relating the normalized mRNA expression values to proteinuria using Pearson correlation.

## Supporting Information

Dataset S1 A molecular signature of proteinuria in glomerulonephritis.
(0.86 MB DOC)
