# Supplementary material for: A Molecular Signature of Proteinuria in Glomerulonephritis
Source: PLoS One. 2010 Oct 18;5(10):e13451. doi: 10.1371/journal.pone.0013451 (PMC2956647; doi:10.1371/journal.pone.0013451)

**A molecular signature of proteinuria in glomerulonephritis**

**Heather N. Reich^*^, David Tritchler‡, Daniel C. Cattran^*^, Andrew M. Herzenberg, Felix Eichinger°, Anissa Boucherot°, Anna Henger°, Celine C. Berthier°, Viji Nair°, Clemens D. Cohen^#^, James W. Scholey^*^ and Matthias Kretzler°**

**Supplementary Data**

Supplementary Table S1. Genes differentially expressed in vitro (p.2)

Supplementary Table S2. Genes differentially expressed in vivo in IgA nephropathy. 49 Albumin-regulated genes differentially expressed in the tubulo-interstitial tissue of patients with IgA nephropathy vs. healthy potential living kidney donors (p.9)

Supplementary Table S3. Genes related to proteinuria. Top genes predictive of or correlated with log-transformed proteinuria in tubulo-interstitial compartment of patients with IgA nephropathy, according to microarray gene-expression measurements (p <0.05) (p.10)

Supplementary Figure S1. Transcriptional network. A transcriptional network including all genes of the 11-mRNA signature was derived based on the co-citation of genes at sentence level in PubMed abstracts by Genomatix BiblioSphere using Natural Language Processing (NLP) (p.11)

**Supplementary Table S1. Genes differentially expressed *in vitro*.**

| **Affymetrix probe set** | **Representative Public ID** | **Gene Symbol** | **Gene Title** |
| --- | --- | --- | --- |
| 212461_at | BF793951 | --- | --- |
| 222281_s_at | AW517716 | --- | --- |
| 209939_x_at | AF005775 | --- | --- |
| 201661_s_at | NM_004457 | ACSL3 | acyl-CoA synthetase long-chain family mem. 3 |
| 209122_at | BC005127 | ADFP | adipose differentiation-related protein |
| 212798_s_at | AK001389 | ANKMY2 | ankyrin repeat and MYND domain containing 2 |
| 202631_s_at | NM_006380 | APPBP2 | amyloid beta precursor protein (cytoplasmic tail) binding protein 2 |
| 202109_at | NM_012402 | ARFIP2 | ADP-ribosylation factor interacting protein 2 (arfaptin 2) |
| 205020_s_at | NM_005738 | ARL4A | ADP-ribosylation factor-like 4A |
| 203404_at | NM_014782 | ARMCX2 | armadillo repeat containing, X-linked 2 |
| 222047_s_at | AI523895 | ARS2 | arsenate resistance protein ARS2 |
| 205047_s_at | NM_001673 | ASNS | asparagine synthetase |
| 203231_s_at | AW235612 | ATXN1 | ataxin 1 |
| 219366_at | NM_020371 | AVEN | apoptosis, caspase activation inhibitor |
| 211379_x_at | AB050855 | B3GALT3 | UDP-Gal:betaGlcNAc beta 1,3-galactosyltransferase, polypeptide 3 |
| 211475_s_at | AF116273 | BAG1 | BCL2-associated athanogene |
| 202326_at | NM_006709 | BAT8 | HLA-B associated transcript 8 |
| 201170_s_at | NM_003670 | BHLHB2 | basic helix-loop-helix domain containing,classB2 |
| 214439_x_at | AF043899 | BIN1 | bridging integrator 1 |
| 217969_at | NM_013265 | C11orf2 | chromosome 11 open reading frame2 |
| 218374_s_at | NM_020374 | C12orf4 | chromosome 12 open reading frame 4 |
| 212992_at | AI935123 | C14orf78 | chromosome 14 open reading frame 78 |
| 218493_at | NM_024571 | C16orf33 | chromosome 16 open reading frame 33 |
| 211563_s_at | AB006572 | C19orf2 | chromosome 19 open reading frame 2 |
| 213989_x_at | AB004853 | C21orf18 | chromosome 21 open reading frame 18 |
| 209301_at | M36532 | CA2 | carbonic anhydrase II |
| 201381_x_at | AF057356 | CACYBP | calcyclin binding protein |
| 214636_at | AA747379 | CALCB | calcitonin-related polypeptide, beta |
| 212971_at | AI769685 | CARS | cysteinyl-tRNA synthetase |
| 205476_at | NM_004591 | CCL20 | chemokine (C-C motif) lIgA nephropathyd 20 |
| 217879_at | AL566824 | CDC27 | cell division cycle 27 |
| 214721_x_at | AL162074 | CDC42EP4 | CDC42 effector protein (Rho GTPase binding) 4 |
| 204510_at | NM_003503 | CDC7 | CDC7 cell division cycle 7 (S. cerevisiae) |
| 201938_at | NM_004642 | CDK2AP1 | CDK2-associated protein 1 |
| 202246_s_at | NM_000075 | CDK4 | cyclin-dependent kinase 4 |
| 202284_s_at | NM_000389 | CDKN1A | cyclin-dependent kinase inhibitor 1A (p21, Cip1) |
| 203973_s_at | NM_005195 | CEBPD | CCAAT/enhancer binding protein (C/EBP), delta |
| 210563_x_at | U97075 | CFLAR | CASP8 and FADD-like apoptosis regulator |
| 213499_at | NM_004366 | CLCN2 | chloride channel 2 |
| 202310_s_at | K01228 | COL1A1 | collagen, type I, alpha 1 |
| **Affymetrix probe set** | **Representative Public ID** | **Gene Symbol** | **Gene Title** |
| 201438_at | NM_004369 | COL6A3 | collagen, type VI, alpha 3 |
| 218072_at | NM_014186 | COMMD9 | COMM domain containing 9 |
| 202469_s_at | AU149367 | CPSF6 | cleavage and polyadenylation specific factor 6, 68kDa |
| 220044_x_at | NM_016424 | CROP | cisplatin resistance-associated overexpressed protein |
| 201904_s_at | BF031714 | CTDSPL | CTD (carboxy-terminal domain, RNA polymerase II, polypeptide A) small phosphatase-like |
| 210764_s_at | AF003114 | CYR61 | cysteine-rich, angiogenic inducer, 61 |
| 203139_at | NM_004938 | DAPK1 | death-associated protein kinase 1 |
| 201440_at | NM_004818 | DDX23 | DEAD (Asp-Glu-Ala-Asp) box polypeptide 23 |
| 48808_at | AI144299 | DHFR | dihydrofolate reductase |
| 212333_at | AL049943 | DKFZP564F0522 | DKFZP564F0522 protein |
| 214889_at | AL080065 | DKFZP564J102 | DKFZP564J102 protein |
| 200881_s_at | NM_001539 | DNAJA1 | DnaJ (Hsp40) homolog, subfamily A, member 1 |
| 213088_s_at | BE551340 | DNAJC9 | DnaJ (Hsp40) homolog, subfamily C, member 9 |
| 203105_s_at | NM_012062 | DNM1L | dynamin 1-like |
| 212838_at | AB023227 | DNMBP | dynamin binding protein |
| 201697_s_at | NM_001379 | DNMT1 | DNA (cytosine-5-)-methyltransferase 1 |
| 209457_at | U16996 | DUSP5 | dual specificity phosphatase 5 |
| 208891_at | BC003143 | DUSP6 | dual specificity phosphatase 6 |
| 201984_s_at | NM_005228 | EGFR | epidermal growth factor receptor (erythroblastic leukemia viral (v-erb-b) oncogene homolog, avian) |
| 201694_s_at | NM_001964 | EGR1 | early growth response 1 |
| 209536_s_at | AF320070 | EHD4 | EH-domain containing 4 |
| 208708_x_at | AL080102 | EIF5 | eukaryotic translation initiation factor 5 |
| 210827_s_at | U73844 | ELF3 | E74-like factor 3 (ets domain transcription factor, epithelial-specific ) |
| 203499_at | NM_004431 | EPHA2 | EPH receptor A2 |
| 203349_s_at | NM_004454 | ETV5 | ets variant gene 5 (ets-related molecule) |
| 221884_at | BE466525 | EVI1 | ecotropic viral integration site 1 |
| 215136_s_at | AL050353 | EXOSC8 | exosome component 8 |
| 219377_at | NM_022751 | FAM59A | family with sequence similarity 59, member A |
| 218539_at | NM_017943 | FBXO34 | F-box protein 34 |
| 210638_s_at | AF176704 | FBXO9 | F-box protein 9 |
| 210950_s_at | BC003573 | FDFT1 | farnesyl-diphosphate farnesyltransferase 1 |
| 218824_at | NM_018215 | FLJ10781 | hypothetical protein FLJ10781 |
| 219450_at | NM_018302 | FLJ11017 | hypothetical protein FLJ11017 |
| 218651_s_at | NM_018357 | FLJ11196 | acheron |
| 218312_s_at | NM_023926 | FLJ12895 | hypothetical protein FLJ12895 |
| 218035_s_at | NM_019027 | FLJ20273 | RNA-binding protein |
| 204420_at | BG251266 | FOSL1 | FOS-like antigen 1 |
| 213524_s_at | NM_015714 | G0S2 | putative lymphocyte G0/G1 switch gene |
| 219539_at | NM_024775 | GEMIN6 | gem (nuclear organelle) associated protein 6 |
| 209276_s_at | AF162769 | GLRX | glutaredoxin (thioltransferase) |
| 218361_at | NM_018178 | GOLPH3L | golgi phosphoprotein 3-like |
| **Affymetrix probe set** | **Representative Public ID** | **Gene Symbol** | **Gene Title** |
| 202453_s_at | NM_005316 | GTF2H1 | general transcription factor IIH, polypeptide 1, 62kDa |
| 201065_s_at | NM_001518 | GTF2I /// GTF2IP1 | general transcription factor II, i /// general transcription factor II, i, pseudogene 1 |
| 201338_x_at | NM_002097 | GTF3A | general transcription factor IIIA |
| 208886_at | BC000145 | H1F0 | H1 histone family, member 0 |
| 211999_at | Z48950 | H3F3B | H3 histone, family 3B (H3.3B) |
| 201145_at | NM_006118 | HAX1 | HS1 binding protein |
| 203821_at | NM_001945 | HBEGF | heparin-binding EGF-like growth factor |
| 209273_s_at | BG387555 | HBLD2 | HESB like domain containing 2 |
| 202814_s_at | AW193511 | HIS1 | HMBA-inducible |
| 215071_s_at | AL353759 | HIST1H2AC | histone 1, H2ac |
| 218280_x_at | NM_003516 | HIST2H2AA | histone 2, H2aa |
| 202708_s_at | NM_003528 | HIST2H2BE | histone 2, H2be |
| 202934_at | AI761561 | HK2 | hexokinase 2 |
| 208808_s_at | BC000903 | HMGB2 | high-mobility group box 2 |
| 202540_s_at | NM_000859 | HMGCR | 3-hydroxy-3-methylglutaryl-Coenzyme A reductase |
| 205822_s_at | NM_002130 | HMGCS1 | 3-hydroxy-3-methylglutaryl-Coenzyme A synthase 1 (soluble) |
| 203665_at | NM_002133 | HMOX1 | heme oxygenase (decycling) 1 |
| 213844_at | NM_019102 | HOXA5 | homeo box A5 |
| 209905_at | AI246769 | HOXA9 | homeo box A9 |
| 221667_s_at | AF133207 | HSPB8 | heat shock 22kDa protein 8 |
| 206976_s_at | NM_006644 | HSPH1 | heat shock 105kDa/110kDa protein 1 |
| 208881_x_at | BC005247 | IDI1 | isopentenyl-diphosphate delta isomerase |
| 202081_at | NM_004907 | IER2 | immediate early response 2 |
| 201631_s_at | NM_003897 | IER3 | immediate early response 3 |
| 212143_s_at | BF340228 | IGFBP3 | insulin-like growth factor binding protein 3 |
| 205207_at | NM_000600 | IL6 | interleukin 6 (interferon, beta 2) |
| 211506_s_at | AF043337 | IL8 | interleukin 8 |
| 210511_s_at | M13436 | INHBA | inhibin, beta A (activin A, activin AB alpha polypeptide) |
| 201627_s_at | NM_005542 | INSIG1 | insulin induced gene 1 |
| 204057_at | AI073984 | IRF8 | interferon regulatory factor 8 /// interferon regulatory factor 8 |
| 201362_at | AF205218 | IVNS1ABP | influenza virus NS1A binding protein |
| 200048_s_at | NM_006694 | JTB | jumping translocation breakpoint /// jumping translocation breakpoint |
| 210119_at | U73191 | KCNJ15 | potassium inwardly-rectifying channel, subfamily J, member 15 |
| 220412_x_at | NM_005714 | KCNK7 | potassium channel, subfamily K, member 7 |
| 219479_at | NM_024089 | KDELC1 | KDEL (Lys-Asp-Glu-Leu) containing 1 |
| 202417_at | NM_012289 | KEAP1 | kelch-like ECH-associated protein 1 |
| 202181_at | NM_014734 | KIAA0247 | KIAA0247 |
| 213424_at | AB020702 | KIAA0895 | KIAA0895 protein |
| 211762_s_at | BC005978 | KPNA2 | karyopherin alpha 2 (RAG cohort 1, importin alpha 1) /// karyopherin alpha 2 (RAG cohort 1, importin alpha 1) |
| 201596_x_at | NM_000224 | KRT18 | keratin 18 |
| **Affymetrix probe set** | **Representative Public ID** | **Gene Symbol** | **Gene Title** |
| 221011_s_at | NM_030915 | LBH | likely ortholog of mouse limb-bud and heart gene /// likely ortholog of mouse limb-bud and heart gene |
| 202068_s_at | NM_000527 | LDLR | low density lipoprotein receptor (familial hypercholesterolemia) |
| 205266_at | NM_002309 | LIF | leukemia inhibitory factor (cholinergic differentiation factor) |
| 219181_at | NM_006033 | LIPG | lipase, endothelial |
| 218263_s_at | NM_021211 | LOC58486 | transposon-derived Buster1 transposase-like protein gene |
| 36711_at | AL021977 | MAFF | v-maf musculoaponeurotic fibrosarcoma oncogene homolog F (avian) |
| 215499_at | AA780381 | MAP2K3 | mitogen-activated protein kinase kinase 3 /// mitogen-activated protein kinase kinase 3 |
| 200769_s_at | NM_005911 | MAT2A | methionine adenosyltransferase II, alpha |
| 209579_s_at | AL556619 | MBD4 | methyl-CpG binding domain protein 4 |
| 200798_x_at | NM_021960 | MCL1 | myeloid cell leukemia sequence 1 (BCL2-related) |
| 221864_at | AW517464 | MGC13024 | hypothetical protein MGC13024 |
| 200899_s_at | NM_012215 | MGEA5 | meningioma expressed antigen 5 (hyaluronidase) |
| 202593_s_at | NM_016641 | MIR16 | membrane interacting protein of RGS16 |
| 204423_at | NM_013255 | MKLN1 | muskelin 1, intracellular mediator containing kelch motifs |
| 221692_s_at | AB049652 | MRPL34 | mitochondrial ribosomal protein L34 /// mitochondrial ribosomal protein L34 |
| 209421_at | U04045 | MSH2 | mutS homolog 2, colon cancer, nonpolyposis type 1 (E. coli) |
| 201761_at | NM_006636 | MTHFD2 | methylenetetrahydrofolate dehydrogenase (NADP+ dependent) 2, methenyltetrahydrofolate cyclohydrolase |
| 210289_at | AB013094 | NAT8 | N-acetyltransferase 8 (camello like) |
| 204823_at | NM_014903 | NAV3 | neuron navigator 3 |
| 203315_at | BC000103 | NCK2 | NCK adaptor protein 2 |
| 200632_s_at | NM_006096 | NDRG1 | N-myc downstream regulated gene 1 |
| 201158_at | AI570834 | NMT1 | N-myristoyltransferase 1 |
| 202679_at | NM_000271 | NPC1 | Niemann-Pick disease, type C1 |
| 209505_at | AI951185 | NR2F1 | Nuclear receptor subfamily 2, group F, member 1 |
| 209121_x_at | M64497 | NR2F2 | nuclear receptor subfamily 2, group F, member 2 |
| 202599_s_at | NM_003489 | NRIP1 | nuclear receptor interacting protein 1 |
| 200790_at | NM_002539 | ODC1 | ornithine decarboxylase 1 |
| 209240_at | AF070560 | OGT | O-linked N-acetylglucosamine (GlcNAc) transferase (UDP-N-acetylglucosamine:polypeptide-N-acetylglucosaminyl transferase) |
| 218304_s_at | NM_022776 | OSBPL11 | oxysterol binding protein-like 11 |
| 212718_at | BF797555 | PAPOLA | poly(A) polymerase alpha |
| 217738_at | BF575514 | PBEF1 | pre-B-cell colony enhancing factor 1 |
| 208396_s_at | NM_005019 | PDE1A | phosphodiesterase 1A, calmodulin-dependent |
| **Affymetrix probe set** | **Representative Public ID** | **Gene Symbol** | **Gene Title** |
| 214129_at | AI821791 | PDE4DIP | Phosphodiesterase 4D interacting protein (myomegalin) |
| 209803_s_at | AF001294 | PHLDA2 | pleckstrin homology-like domain, family A, member 2 |
| 201928_at | AA194254 | PKP4 | plakophilin 4 |
| 211668_s_at | K03226 | PLAU | plasminogen activator, urokinase /// plasminogen activator, urokinase |
| 210845_s_at | U08839 | PLAUR | plasminogen activator, urokinase receptor |
| 212100_s_at | Z93241 | POLDIP3 | polymerase (DNA-directed), delta interacting protein 3 |
| 218016_s_at | NM_018119 | POLR3E | polymerase (RNA) III (DNA directed) polypeptide E (80kD) |
| 203966_s_at | NM_021003 | PPM1A | protein phosphatase 1A (formerly 2C), magnesium-dependent, alpha isoform /// protein phosphatase 1A (formerly 2C), magnesium-dependent, alpha isoform |
| 202187_s_at | NM_006243 | PPP2R5A | protein phosphatase 2, regulatory subunit B (B56), alpha isoform |
| 219515_at | NM_020228 | PRDM10 | PR domain containing 10 |
| 203089_s_at | NM_013247 | PRSS25 | protease, serine, 25 |
| 208777_s_at | AF001212 | PSMD11 | proteasome (prosome, macropain) 26S subunit, non-ATPase, 11 |
| 205194_at | NM_004577 | PSPH | phosphoserine phosphatase |
| 216306_x_at | X62006 | PTBP1 | polypyrimidine tract binding protein 1 |
| 211756_at | BC005961 | PTHLH | parathyroid hormone-like hormone /// parathyroid hormone-like hormone |
| 218948_at | AL136679 | QRSL1 | glutaminyl-tRNA synthase (glutamine-hydrolyzing)-like 1 |
| 74694_s_at | AA907940 | RABEP2 | rabaptin, RAB GTPase binding effector protein 2 |
| 213313_at | AI922519 | RABGAP1 | RAB GTPase activating protein 1 |
| 204478_s_at | NM_002871 | RABIF | RAB interacting factor |
| 201046_s_at | NM_005053 | RAD23A | RAD23 homolog A (S. cerevisiae) |
| 212646_at | D42043 | RAFTLIN | raft-linking protein |
| 202297_s_at | AF157324 | RER1 | RER1 retention in endoplasmic reticulum 1 homolog (S. cerevisiae) |
| 204337_at | AL514445 | RGS4 | regulator of G-protein signalling 4 |
| 204268_at | NM_005978 | S100A2 | S100 calcium binding protein A2 |
| 212845_at | AB028976 | SAMD4 | sterile alpha motif domain containing 4 |
| 209146_at | AV704962 | SC4MOL | sterol-C4-methyl oxidase-like |
| 202071_at | NM_002999 | SDC4 | syndecan 4 (amphiglycan, ryudocan) |
| 203528_at | NM_006378 | SEMA4D | sema domain, immunoglobulin domain (Ig), transmembrane domain (TM) and short cytoplasmic domain, (semaphorin) 4D |
| 208940_at | AI885670 | SEPHS1 | Selenophosphate synthetase 1 |
| 202628_s_at | NM_000602 | SERPINE1 | serine (or cysteine) proteinase inhibitor, clade E (nexin, plasminogen activator inhibitor type 1), member 1 |
| 33323_r_at | X57348 | SFN | stratifin |
| 208673_s_at | AF107405 | SFRS3 | splicing factor, arginine/serine-rich 3 |
| 201129_at | NM_006276 | SFRS7 | splicing factor, arginine/serine-rich 7, 35kDa |
| **Affymetrix probe set** | **Representative Public ID** | **Gene Symbol** | **Gene Title** |
| 206872_at | NM_005074 | SLC17A1 | solute carrier family 17 (sodium phosphate), member 1 |
| 209681_at | AF153330 | SLC19A2 | solute carrier family 19 (thiamine transporter), member 2 |
| 209610_s_at | BF340083 | SLC1A4 | solute carrier family 1 (glutamate/neutral amino acid transporter), member 4 |
| 201920_at | NM_005415 | SLC20A1 | solute carrier family 20 (phosphate transporter), member 1 |
| 205074_at | NM_003060 | SLC22A5 | solute carrier family 22 (organic cation transporter), member 5 |
| 217122_s_at | AL031282 | SLC35E2 | solute carrier family 35, member E2 |
| 209921_at | AB040875 | SLC7A11 | solute carrier family 7, (cationic amino acid transporter, y+ system) member 11 |
| 216604_s_at | AL365343 | SLC7A8 | Solute carrier family 7 (cationic amino acid transporter, y+ system), member 8 |
| 201417_at | AL136179 | SOX4 | SRY (sex determining region Y)-box 4 |
| 204011_at | NM_005842 | SPRY2 | sprouty homolog 2 (Drosophila) |
| 221489_s_at | W48843 | SPRY4 | sprouty homolog 4 (Drosophila) |
| 209218_at | AF098865 | SQLE | squalene epoxidase |
| 38766_at | AB002307 | SRCAP | Snf2-related CBP activator protein |
| 218140_x_at | NM_021203 | SRPRB | signal recognition particle receptor, B subunit |
| 205542_at | NM_012449 | STEAP1 | six transmembrane epithelial antigen of the prostate 1 |
| 219686_at | NM_018401 | STK32B | serine/threonine kinase 32B |
| 202951_at | BE048506 | STK38 | serine/threonine kinase 38 |
| 209238_at | BE966922 | STX3A | syntaxin 3A |
| 201263_at | NM_003191 | TARS | threonyl-tRNA synthetase |
| 212330_at | R60866 | TFDP1 | transcription factor Dp-1 |
| 203588_s_at | BG034328 | TFDP2 | transcription factor Dp-2 (E2F dimerization partner 2) |
| 212910_at | W19873 | THAP11 | THAP domain containing 11 |
| 203887_s_at | NM_000361 | THBD | thrombomodulin |
| 213301_x_at | AL538264 | TIF1 | transcriptional intermediary factor 1 |
| 209386_at | AI346835 | TM4SF1 | transmembrane 4 L six family member 1 |
| 202857_at | NM_014255 | TMEM4 | transmembrane protein 4 |
| 202687_s_at | U57059 | TNFSF10 | tumor necrosis factor (lIgA nephropathyd) superfamily, member 10 /// tumor necrosis factor (lIgA nephropathyd) superfamily, 10 |
| 214550_s_at | AF145029 | TNPO3 | transportin 3 |
| 208901_s_at | J03250 | TOP1 | topoisomerase (DNA) I |
| 203567_s_at | AU157590 | TRIM38 | tripartite motif-containing 38 |
| 219474_at | NM_024616 | TTMP | TPA-induced transmembrane protein |
| 209118_s_at | AF141347 | TUBA3 | tubulin, alpha 3 |
| 201714_at | NM_001070 | TUBG1 | tubulin, gamma 1 |
| 201009_s_at | AI439556 | TXNIP | thioredoxin interacting protein |
| 201588_at | NM_004786 | TXNL1 | thioredoxin-like 1 |
| 202589_at | NM_001071 | TYMS | thymidylate synthetase |
| 46270_at | AL039447 | UBAP1 | ubiquitin associated protein 1 |
| 200684_s_at | AI819709 | UBE2L3 | ubiquitin-conjugating enzyme E2L 3 |
| 203234_at | NM_003364 | UPP1 | uridine phosphorylase 1 |
| **Affymetrix probe set** | **Representative Public ID** | **Gene Symbol** | **Gene Title** |
| 201672_s_at | NM_005151 | USP14 | ubiquitin specific protease 14 (tRNA-guanine transglycosylase) |
| 218806_s_at | AF118887 | VAV3 | vav 3 oncogene |
| 210285_x_at | BC000383 | WTAP | Wilms tumor 1 associated protein |
| 210301_at | U06117 | XDH | xanthine dehydrogenase |
| 202778_s_at | NM_003453 | ZNF198 | zinc finger protein 198 |
| 204937_s_at | NM_016325 | ZNF274 | zinc finger protein 274 |

**Supplementary Table S2. Genes differentially expressed in IgA nephropathy vs. control**

3A. Genes Upregulated in IgA nephropathy vs. Control

**Symbol Description**

ANKMY2 ankyrin repeat and MYND domain containing 2

COL1a1 collagen, type I, alpha 1

COL6A3 collagen, type VI, alpha 3

GTF2I general transcription factor II, i

HMGB2 high-mobility group box 2

HOXA9 homeo box A9

KDELC1 KDEL (Lys-Asp-Glu-Leu) containing 1

LOC58486

MGEA5 meningioma expressed antigen 5 (hyaluronidase)

SEPHS1 selenophosphate synthetase 1

SFRS7 splicing factor, arginine/serine-rich 7, 35kDa

SLC35E2 solute carrier family 35, member E2

STEAP1 six transmembrane epithelial antigen of the prostate 1

STK38 serine/threonine kinase 38

TARS threonyl-tRNA synthetase

TYMS thymidylate synthetase

UBE2L3 ubiquitin-conjugating enzyme E2L 3

3B. Genes downregulated in IgA nephropathy vs. control

**Symbol Description**

ADFP adipose differentiation-related protein

BHLHB2 basic helix-loop-helix domain containing, class B, 2

CDKN1A cyclin-dependent kinase inhibitor 1A (p21, Cip1)

CEBPD CCAAT/enhancer binding protein (C/EBP), delta

CYR61 cysteine-rich, angiogenic inducer, 61

DUSP5 dual specificity phosphatase 5

DUSP6 dual specificity phosphatase 6

EGR1 early growth response 1

ELF3 E74-like factor 3 (ets domain transcription factor, epithelial-specific )

EPHA2 EPH receptor A2

FOSL1 FOS-like antigen 1

G0S2 G0/G1switch 2

HBEGF heparin-binding EGF-like growth factor

HMGCR 3-hydroxy-3-methylglutaryl-Coenzyme A reductase

IER2 immediate early response 2

IER3 immediate early response 3

IL6 interleukin 6 (interferon, beta 2)

INHBA inhibin, beta A (activin A, activin AB alpha polypeptide)

LDLR low density lipoprotein receptor (familial hypercholesterolemia)

LIF leukemia inhibitory factor (cholinergic differentiation factor)

LIPG lipase, endothelial

MAFF v-maf musculoaponeurotic fibrosarcoma oncogene homolog F (avian)

MAP2K3 mitogen-activated protein kinase kinase 3

MCL1 myeloid cell leukemia sequence 1 (BCL2-related)

PHLDA2 pleckstrin homology-like domain, family A, member 2

SAMD4 sterile alpha motif domain containing 4

SERPINE1 serpin peptidase inhibitor, PAI-1

SLC19A2 solute carrier family 19 (thiamine transporter), member 2

SLC20A1 solute carrier family 20 (phosphate transporter), member 1

SPRY2 sprouty homolog 2 (Drosophila)

THBD thrombomodulin

TTMP chromosome 3 open reading frame 52

# Supplementary Table S3. Genes predictive of or correlated with proteinuria

| ARFIP2 | ELF3 | KDELC1 | PKP4 | TM4SF1 |
| --- | --- | --- | --- | --- |
| CACYBP | ETV5 | KPNA2 | PSMD11 | TMEM4 |
| CDK2AP1 | GLRX | KRT18 | SERPINE1 | TNFSF10 |
| CDKN1A | HBEGF | MAFF | S100A2 | TRIM38 |
| COL1A1 | HBLD2 | MBD4 | SAMD4 | TUBA3 |
| COL6A3 | HMGB2 | MCL1 | SFN | TXNL1 |
| CYR61 | HMGCS1 | MIR16 | SFRS3 | TYMS |
| DNAJC9 | HMOX1 | MTHFD2 | SLC19A2 | UPP1 |
| DNMT1 | HSPB8 | NDRG1 | SOX4 | USP14 |
| DUSP6 | IER3 | PBEF1 | STEAP1 | ZNF274 |
| EGR1 | IRF8 | PHLDA2 | TARS | ZNF447 |

Supplementary Figure S1.


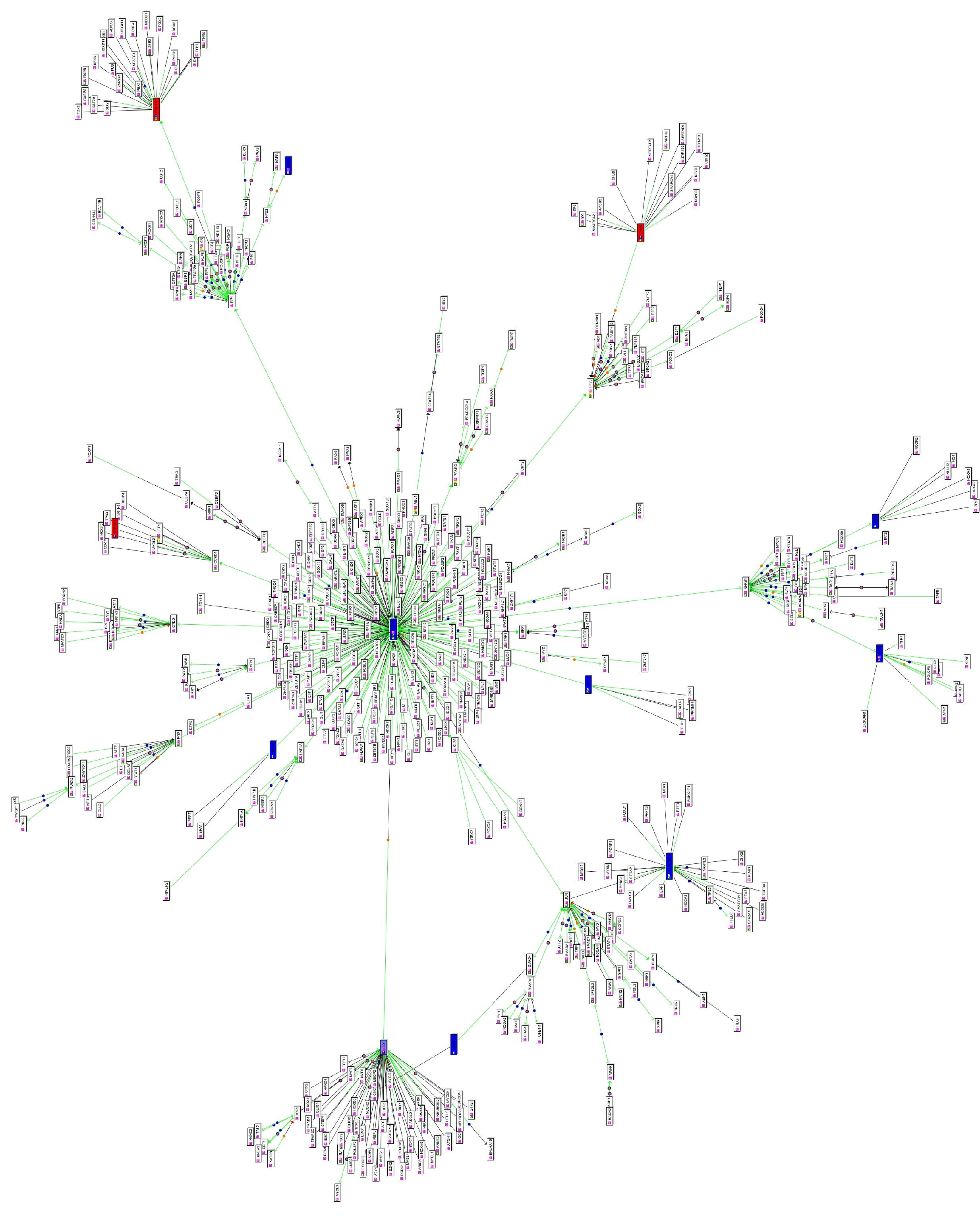

Supplement: Dataset S1 — A molecular signature of proteinuria in glomerulonephritis. (0.86 MB DOC) [file pone.0013451.s001.doc]
